# Supplementary material for: Origami-based integration of robots that sense, decide, and respond
Source: Nat Commun. 2023 Apr 3;14:1553. doi: 10.1038/s41467-023-37158-9 (PMC10070436; doi:10.1038/s41467-023-37158-9)
Supplement: Supplementary file 1 — Supplementary Information [file 41467_2023_37158_MOESM1_ESM.pdf]

Supplementary Information

Origami-based integration of robots that sense, decide, and respond

Wenzhong Yan,[1*] Shuguang Li,[2,3] Mauricio Deguchi,[1]

Zhaoliang Zheng,[4] Daniela Rus,[2] and Ankur Mehta[4]

[1]Mechanical and Aerospace Engineering Department, UCLA, USA.

[2]Computer Science and Artificial Intelligence Laboratory, MIT, USA.

[3]Department of Mechanical Engineering, Tsinghua University, P.R. China.

[4]Electrical and Computer Engineering Department, UCLA, USA.

[*]To whom correspondence should be addressed; E-mail: wzyan24@g.ucla.edu

Supplementary Discussion:

Our OMS and logic gates use electrical signals as both inputs and outputs; this architecture makes it possible to use the output to control any electrically or thermally driven actuators (e.g., shape memory alloy actuators (*1*), conductive liquid crystal elastomers (LCE) (*2*), etc.), making them suitable for integrating into a wide range of robots and machines. The system can function equally well under AC signals; though not demonstrated, the core OMS is driven by the current magnitude and is thus independent of its direction of flow. For example, an output AC signal can be directly used to drive an electromagnetic actuator from a comparable supply. For pure mechanical logic networks, the number of inputs that an output signal can drive (i.e., fan-out) is often very small, which is restricted by the limited number of accessible interactions between gates, high damping, and other losses (*3*). Our proposed architecture combined with the nature of low resistance of logic gates vastly enlarges the fan-out, which could potentially increase the overall scope of the resulting computing networks and thus the design space of our proposed system.

Our OMS-based mechanism provides opportunities for adopting other actuation systems, which could lead to broader applications. As stated above, we use the same type of signal for both the inputs and outputs of OMS-based devices. The output signals from previous gates can be directly fed as the inputs of the downstream gates without additional interfacing components. Thus, we have two main requirements for actuators in our system. 1) They can be directly activated by signals of interest. For instance, CSCP actuators can be electrically driven. 2) Actuators are capable of exerting sufficient displacement and force to toggle the bistable mechanism. Taking shape memory alloy (SMA) actuators as an example, they can be electrically activated and generate large linear contractions, which makes them suitable for our systems. Actuators, like ionic polymer-metal composites, might not be competent due to their small output force. Other electrically driven actuators, e.g., conductive LCEs are also viable. Another common ac-

tuation of interest is pneumatic (or fluidic). For instance, linear contracting pneumatic actuators can be used to activate the bistable beam (*4*). Accordingly, we need to use pneumatic current as a signal instead of electricity (*5*). Otherwise, we could build hybrid origami logic systems for specific applications that require different input and output signals, although additional interfacing components are required for cascaded circuits. For example, we can create origami logic with pneumatic inputs but electrical outputs similar to Ref. (*6*). Alternative actuators, e.g., light-driven LCEs, may be adopted into our system to build more hybrid origami logic and autonomous machines.

The proposed devices have been tested to robustly function for the order of hundreds of repetitions, which is sufficient for all of the demonstrations in this paper. Based on our previous work (*7*) that conducted a lifecycle test on a self-sustained oscillator that shares the same materials and physical processes, these devices could presumably work at least thousands of times with marginal degradation in performance; failure largely happened because of the thermal instability of CSCP actuators under cyclic heating/cooling. Extending the durability of origami devices is out of the scope of this paper; the feasibility of long-time operation still needs to be validated experimentally for further exploration. Alternately, this issue can be addressed by replacing the CSCP actuators with known long-life mechanisms, e.g., dielectric elastomer actuators (DEA), although that requires a high working voltage (*8*).

Constructing logic gates and robots with bistable beams enables easy memory and state storage, which allows energy saving since only switching between states requires power. Although it is favorable for information storage, this bistability-based architecture requires a reset after each computational execution. Instead, using monostable beams with snap-through (the beams will snap back to their stable position if the applied force is removed) can enable self-reset at the cost of constant operating power. The self-reset allows continuous operation and thus less external interference for simple circuits (*5*). However, the fabrication of a monostable beam

using folding-based methods is much more challenging due to the high sensitivity to its geometry parameters and boundary conditions (*9*). Therefore, the trade-off between two different architectures needs to be considered for specific tasks.

The delay of a single OMS-based gate is essentially determined by the time needed for the contraction of the CSCP actuator and the snap-through of the bistable beam. Presumably, we could largely shorten the response time of the actuator by increasing the supply voltage while increasing the snap-through speed by adopting a shorter, stiffer, or lightweight beam, or increasing the thickness of the beam (more discussion can be found in Supplementary Note 7 and Supplementary Note 8). However, it is very challenging to further reduce the delay to be comparable with that of semiconductor-based devices, which is also not the goal of this work. Therefore, our OMS-based devices are mainly suitable for speed-insensitive applications. Moreover, our origami-based process provides alternative approaches to generate more complicated circuits. For instance, a self-sustained oscillator can be directly created instead of combining several OMS-based gates (*7*). Other more complex functionalities could presumably be built in a similar manner.

Although our origami components and devices, such as logic gates, are less performant than conventional semiconductor counterparts in terms of speed, density, and energy efficiency, our OMS information processing architecture demonstrates advantages for certain applications without a power constraint. For example, the OMS, sensors, logic gates, and origami robots are built of non-rigid, inexpensive sheet materials (polyester) and conductive sewing threads through origami-inspired folding. This approach allows integrated design and rapid fabrication for accessible, low cost, and potentially disposable designs (*10*). The resulting integrated systems are semiconductor-free, and nonmagnetic, enabling practical applications in extreme environments (e.g., with high magnetic or radiation fields). Meanwhile, our folding-based compliant devices and robots are easier to build and integrate with compared with soft-bodied

polymer counterparts, which typically involve multi-stage molding (or printing) and sealing processes (*5, 6, 11*). The folding-based method for creating robots is engaging and unintimidating, making it desired for applications in the toy industry and education (*10*). The low cost of constituent materials and simple equipment requirements further make our method more accessible for resource-constrained students.

Currently, we have realized up to two levels of cascaded combinational logic; our modularized design of fundamental gates (NOT, AND, and OR) allows us to compose arbitrarily complex gates through further cascading using geometric layout and wiring. However, to broaden the scope of our origami digital logic for autonomous robots, this universal functionality must be augmented by three additional research contributions: i) **Fabrication**. Although our current folding-based process makes it easy to prototype single units, it is limited for high-throughput creation. Solutions for at-scale manufacturing of origami structures include automating self-folding (*12*) through careful materials selection (*13*). ii) **Sizing**. We have currently designed centimeter-scale devices. Though a nominal decrease in size might be possible through further engineering optimization, a several-fold reduction in characteristic dimension—on par with other scaled 2D printable techniques such as pop-up book MEMS (*14*) or direct printing of actuators (*15*)—would practically enable much more complex logic. iii) **Design automation**. With the increasing number of logic gates in more complicated circuits, the design and implementation of desired functionalities become onerous; this could also result in inefficiencies due to unoptimized circuits and their wiring. Computational design tools (e.g., (*16*)) have been used to assist in the generation of manufacturable drawings from functional specifications.

Our work lays the groundwork for future research aimed at creating autonomous robots purely through folding without post-installation. Currently, parts of components (e.g., actuators) still need to be installed after folding processes. Therefore, it is vital to develop versatile 2D composites that can tightly integrate necessary functional materials to complete essential

functionalities, including sensing, computing, and actuation. Pop-up book MEMS is a promising method to embed necessary functionalities into patterned laminates that can be folded to construct autonomous machines. Another encouraging direction is 3D printing, especially multimaterial direct-ink-writing and embedded 3D printing. Thus, multifunctional 2D precursors can be directly created either through lamination or printing and then folded into reprogrammed configurations with desired functionalities without post-installation.

To create untethered autonomous robots fully through origami cut-and-fold, we can incorporate thin film lithium batteries by embedding them into origami structures (*17–19*). These batteries can provide both load-bearing and energy storage and easily be built through 2D fabrication without requiring post-installation. We can also replace the DC motors with origami oscillators (*7*) to generate periodical motions which are essential for the autonomy of robots, especially for locomotion. This would lead to advances in developing untethered intelligent systems of natural counterparts purely through origami-inspired cut-and-fold.

Additional capabilities such as clocking logic and alternate sensing modalities can be achieved within our proposed origami paradigm, although replicating semiconductor-based electronic computers and machines is not the underlying goal of our research. We can create a clock by using origami oscillators that generate alternating signals from a constant voltage supply as presented in our previous work (*20*). Integrating this clock enables sequential logic and higher autonomy for origami machines. Sensors that perceive different signals allow a broader range of interactions with humans and the environment. One way they can be constructed is by replacing the CSCP actuators on the bistable (or monostable) beam of OMS with stimuli-responsive materials (Supplementary Fig.23); thus the corresponding stimuli can be transduced into an output voltage signal. For example, the OMS can be modified as an optical sensor by substituting the CSCP actuators with photo-responsive liquid crystal elastomers (*2*).

Fabrication of Devices:

Supplementary Note 1 Fabrication of OMS and logic gates

The OMS and logic gates share the same fabrication process (Supplementary Fig.3). Here we use an origami NOT as an example to demonstrate the fabrication method; the detailed creation of each logic can be found in Supplementary Fig.5-9. This also similarly corresponds to that of the crawler (Supplementary Fig.13), flytrap-inspired robot (Supplementary Fig.15), and origami wheeled car (Supplementary Fig.18). The fabrication of the NOT is mainly done in three stages: (i) origami cut-and-fold (with contact pads attached), (ii) attaching copper tape circuit traces, and (iii) assembly of CSCP actuators.

The NOT gate is firstly constructed by patterning a flexible, polyester film (DuraLar^{TM}, Grafix Plastics) with a cutting machine (Silhouette CAMEO 2, Silhouette America, Inc.). The 2D fabrication pattern of the NOT is shown in Supplementary Fig.4A, where the red and blue dashed lines indicate mountain and valley folds, respectively. Before folding, the contact pads are attached to the tabs; otherwise, it would be very challenging to fix them on the buckled beam (Supplementary Fig.4C). The contact pads were partnered in such a fashion that the lower extruding rectangular parts, were facing away from one another. After partnering them, the smaller, T-shaped piece was attached to the thin, center, stick-like, cut out of the beam and the top edges of the T were wrapped around to ensure adhesion. Next, the z-shaped piece was directly attached to the beam with its upper rectangular piece being placed underneath the previous T-shaped pad, creating electrical contact between the two surfaces. Thus the two pads only contact when the bistable beam is at a certain stable state.

The origami frame is then folded from a planar base and various out-of-plane structures; these structures can be visualized in Supplementary Fig.4B, indicated by the two supports along the boundary and the beam support through the center. These structures are folded in a weaving manner, coming up through a slot from below the base and folding back down through the

various support slots to form the arches. By using origami features as connectors, we can minimize the resources required to assemble the devices. More fabrication details of this class of origami devices can be found in (*21*).

Secondly, we place the copper tape circuit traces (see Supplementary Fig.4D) on the base of the origami framing (Supplementary Fig.4B) while connected with contact pads through copper wires. The widths of copper traces could vary since they have very high conductivity compared with other electrical components, e.g., CSCP actuators. For convenience, we use about 3mm traces. Although it would require extra effort and care during folding, these copper traces can also be laminated onto the PET sheet before folding. For simplicity, we chose to install them after folding. Lastly, we install the actuator onto the resulting structure. One terminal of the CSCP actuator was fixed to the beam by using a piece of heat-shrink tubing (7496K81, Insultab) on either side. The other terminal of the actuator was similarly fixed to the support structure through another heat-shrink tube. Each of the exterior ends of the actuators was then connected to the circuit traces using thin copper wires, completing the electrical connection of the circuit.

Supplementary Note 2 Fabrication of the flytrap-inspired robot

The fabrication of the flytrap-inspired robot and the crawler robot shares a similar process to that of the origami NOT described above. The robot contains an AND gate (without reset mechanism) at its central base as shown in Supplementary Fig.15A. Its corresponding folding pattern is shown in Supplementary Fig.15C. The outer leaves were then attached to the central switch through the leaf-base connections (see Supplementary Fig.15A and B). These connections use double rib tabs, similar to those previously mentioned for the various beams and supports, to support the opening and closing of the leaves. A touch sensor, based on the bistable switch, is formed on the center of each leaf by adhering the contact pads shown in Supplementary Fig.15C to both bistable beams. The outputs of the two sensors are connected to the two inputs of the

AND gate (see Fig.5b). Then, the actuators are threaded through the holes indicated in Supplementary Fig.15C and tethered to the bottom platform (in green) and the support structure on the leaf itself, using shrink tubes. When the actuators contract, the leaves rotate along with the hinges on the connections to capture living ‘prey’. The complete circuit diagram of the robot can be found in Supplementary Fig. 17.

Supplementary Note 3 Fabrication of the legged robot

The origami crawler consists of body, legs, antennae (with embedded touch sensors), origami controller, and a 3.7-V Lithium battery. (i) The body was firstly constructed by folding in accordance with the manually-designed patterns shown in Supplementary Fig.13; the various components were then mounted together into their designated slots, completing the crawler’s body. (ii) Circuit traces, including contact pads, were then cut and placed on the body (see Supplementary Fig.14 for the detailed circuit diagram). The sensing antenna function by completing one path of the circuit when contact is made against a surface. Consistent, sustained contact is necessary in order to trigger the corresponding reversal mechanism; thus magnets were placed underneath the surface of the upper body and just in front of the antenna. These magnets guarantee a solid connection between the two contact pads, once a sufficient force has been applied for the magnetic force to bring the antenna to the body. Alternative methods were explored, such as that of a bistable beam, but much larger forces were required to trigger such switches. (iii) One CSCP actuator was incorporated in nearly the same manner as described for the NOT gate with a single key distinction, only one support is required since the beam itself can act as an attachment point with manual reset. Another CSCP actuator was similarly attached to the DPDT switch to alter the state of the switch. (iv) Next, the various electronic components including a battery, a power switch, an 8-ohm resistor, and two DC motors (FS90R, Pololu Corporation) were embedded into the circuit as shown in Supplementary Fig.14. Much of the

wiring is taken care of by the printed circuit trace pattern with these various components being implemented in series with the circuit, in the empty middle layer of the crawler. The active legs were attached to the output shafts of the motors to complete the assembly.

Supplementary Note 4 Fabrication of the origami wheeled car

The origami wheeled car consists of three major parts: one origami body, one memory disc, and two corrugated wheels (see Fig. 7a). The body of the car was folded from a PET sheet with the 2D pattern shown in Supplementary Fig.18A. The motor frame is designed to hold the DC motors (FS90R, Pololu Corporation) that are used to drive the wheels. To improve the stiffness of the wheels and the robustness of locomotion, we adopted a corrugated wheel design (*22*). One rubber band was attached to each wheel to increase the friction to prevent sliding for stable locomotion. The key component of the car is the memory disc with its corresponding 2D pattern in Supplementary Fig.18C; it includes supporting disc, bistable memory bits, and associates. The supporting disc was cut from a 1.5mm-thick clear acrylic sheet by a laser cutter (Speedy 300 flexx, Trotec). The basic memory bits sharing a similar design as OMS were fixed on the supporting disc through customized beam supports; this modular design can reduce fabrication difficulty. The information (voltage) of memory bits are read by the rotating head driven by a gearbox motor (HP 6V, Pololu Corporation, attached on the disc through the center motor slot); the sliding brushes on the read head extracted the signal through the electrical contact with the copper sliding rings (see Supplementary Fig.18D). We also specially designed a two-channel slip ring to enable the information transmission from the rotating read head to stationary motors on the car. The detailed circuit diagram of the origami car is present in Supplementary Fig.19, though only one composite memory bit (including two basic bistable memory bits) is shown in Supplementary Fig.19A.

Characterization of Components:

Supplementary Note 5 Characterization of the CSCP actuator

There were two properties, thermal coefficient, c_T and stiffness k, of the CSCP actuator that needed to be characterized. The detailed parameters of the tested actuator sample are listed in Supplementary Table 1. To obtain c_T, we heated the actuator through Joule heating with a DC power supply (TP-3003D-3, Kaito Electronics, Inc.). When the actuator's temperature reached a steady state, the temperature and its corresponding generated force read from the Mechanical Test Systems (MultiTest, Mecmesin) were recorded. In the same manner, we changed the supply power, and thus temperature and recorded corresponding exerted force to plot the temperature-force curve, as shown in Supplementary Fig.20A. By repeating the same test three times on two samples, we could fit the data with a linear curve. The curve's mean slope is the thermal coefficient, c_T, approximated as 2.18×10^{-2} N/°C (*23*).

k is determined through the slope of the force-displacement curve (*23*) produced by using the Mechanical Test Systems. A K-type thermal couple (CT-QB-K-0.1, PerfectPrime) and a thermometer (COMINHKPR146086, Leaton) were attached to the actuator using cyanoacrylate glue (Krazy glue, Elmer's Product, Inc.). The force-displacement curve has a slope of around 0.39, which means the mean stiffness of the 50-mm-long actuator is 0.39 N/mm (Supplementary Fig.20B).

Supplementary Note 6 Characterization of the bistable beam

The specific geometry parameters of the bistable beam testing samples are listed in Supplementary Table 1. During the experiment, the Mechanical Test Systems (MultiTest, Mecmesin) can directly export the force-displacement curve of the bistable beam. We repeated the test three times and plot the force-displacement curve with a calculated standard deviation in light red color (see Supplementary Fig.21). This curve indicates the values of the w_{rise}, w_{cr}, and F_{cr} are

-2.12 mm, -0.83 mm, and 0.35 N. Note that the values of w_{rise}, w_{cr}, and F_{cr}, are converted to the coordinate system as shown in Supplementary Fig.22A.

Theory and Modeling:

Supplementary Note 7 Analytical modeling for the gate delay of the OMS

The schematic of the simplified OMS (actuator-bistable beam system) is denoted in Supplementary Fig.22. Hence, the positive direction of the selected coordinate is pointing upwards. The beam's length, width, and thickness are labeled as L_0, b, and h, respectively. L represents the span of the buckled beam. Using the Euler-Bernoulli beam model, the actuator-bistable beam system can be modeled by the governing equation, Supplementary Equation (1) with its boundary condition, Supplementary Equation (2), and initial condition, Supplementary Equation (3) (*24*):

$$EI\frac{\partial^4 w}{\partial x^4} + P\frac{\partial^2 w}{\partial x^2} - \frac{EA}{2L}\frac{\partial^2 w}{\partial x^2}\int_0^L (\frac{\partial w}{\partial x})^2 dx + m\frac{\partial^2 w}{\partial t^2} + c\frac{\partial w}{\partial t} = F(w,t)\delta(x-0.5L) \tag{1}$$

$$w(0,t) = \frac{\partial w}{\partial x}(0,t) = 0, \quad w(L,t) = \frac{\partial w}{\partial x}(L,t) = 0 \tag{2}$$

$$w(x,0) = \frac{w_{\text{rise}}}{2}[1 - cos(\frac{2\pi x}{L})] \tag{3}$$

where $F(w,t)\delta(x-0.5L)$ (δ is the Dirac delta function) represents a point force $F(x,w,t)$ applied on the beam at the position $x = 0.5L$ (center point C) as an equivalent distributive load. We denote the mass per unit length, Young's modulus, damping coefficient, axial loading, second moment, and cross-sectional area of the beam as m, E, c, P, I ($I = bh^3/12$), and A ($A = bh$), respectively. The beam's midpoint has an initial rise of w_{rise}. The force exerted by the actuator is the following (*23*):

$$F(w,t) = k(w - w_{\text{rise}}) + b_{\text{a}}\dot{w} + c_{\text{T}}[T(t) - T_0] \tag{4}$$

where k and b_{a} represent the mean stiffness and mean damping of the actuator, respectively. Due to the special coordinate, the value of k is negative. The actuator's temperature is $T(t)$, while T_0

is the room temperature, and c_{T} is the mean slope that translates the temperature rise to force. Typically, the actuator's velocity before snap-through is minimal, under 3×10^{-3} m/s (see Fig. 2c), enabling us to dismiss the effect of damping. Originating from a first-order thermoelectric model, the actuator's temperature rise is described with Supplementary Equation (5)

$$T(t) = \frac{U^2}{\lambda R}(1 - e^{-\frac{\lambda}{C_{\text{th}}}t}) + T_0 \tag{5}$$

where λ, C_{th}, and R are the absolute thermal conductivity, thermal mass, and resistance of the actuator, respectively. While the voltage across the actuator is U.

Based on the quasi-static assumption that the dynamics of the actuator dictate the time-resolved behavior of the integrated system (*25*), we can reduce the model by eliminating the time derivative terms in Supplementary Equation (1) to obtain a simplified ODE. Thus, we can perform discretized time steps and solve for the displacement of point C at any point in time, during the actuation process. The resulting ODE describes the displacement at a specific time point t_{i}, as shown in Supplementary Equation (6) and Supplementary Equation (7).

$$EI\frac{d^4 w_{\mathrm{i}}}{dx^4} + P\frac{d^2 w_{\mathrm{i}}}{dx^2} - \frac{EA}{2L}\frac{d^2 w_{\mathrm{i}}}{dx^2}\int_0^L (\frac{dw_{\mathrm{i}}}{dx})^2 dx = F(w)\delta(x - 0.5L) \tag{6}$$

$$F(w_{\mathrm{i}}, t_{\mathrm{i}}) = k(w_{\mathrm{i}} - w_{\text{rise}}) + c_{\mathrm{T}}[T(t_i) - T_0] \tag{7}$$

Therefore, we are able to separate the system and consider the force exerted from the actuator as a series of time-resolved inputs of Supplementary Equation (6). Supplementary Fig.22 describes this process, in which the force-displacement curve of the bistable beam is depicted by the blue curve while that of the actuator is captured by the straight lines. At its initial state (t=t_0), the actuator's force-displacement curve (black line) intersects with that of the bistable beam at (w=w_{rise}, F=0). Over time, the actuator's temperature rises, causing its force-displacement curve to shift upward, with the intersection of the two curves approaching the critical point (w=w_{cr}, F=F_{cr}). As a result, the bistable beam snaps through. Thus, we know that the once

t_{snap} satisfies the equation:

$$F_{\text{cr}} = k(w_{\text{cr}} - w_{\text{rise}}) + c_{\text{T}}[T(t_{\text{snap}}) - T_0] \tag{8}$$

We can solve for Supplementary Equation (8) by combining with Supplementary Equation (5), to have the expression of the actuation period, $T_{\text{actuation}}$ (= t_{snap}) as follows:

$$T_{\text{actuation}} = -\frac{C_{\text{th}}}{\lambda} ln\{1 - \frac{\lambda R}{c_{\text{T}} U^2}[F_{\text{cr}} - k(w_{\text{cr}} - w_{\text{rise}})]\} \tag{9}$$

According to the definition of gate delay, this $\text{T}_{\text{actuation}}$ must be calculated when the opposite (reset) CSCP actuator is fully cooled down to ambient temperature. For Supplementary Equation (9) to be meaningful, we must force the value of the expression inside Logarithmic function to always remain positive, then we have Supplementary Equation (10).

$$V_{supp} > \sqrt{\frac{\lambda R}{c_T}[F_{snap-thru} - k(w_{snap-thru} - w_{rise})]} \tag{10}$$

V_{supp} is the supplied electrical voltage across the actuator with a lower bound for snap-through defined by Supplementary Equation (10).

This expression serves to define the exclusive lower bound of the current necessary to cause the snap-through for a specific switch. Otherwise, the bistable bean will remain somewhere between w_{rise} and w_{cr} (see Supplementary Fig.1 and Supplementary Fig.22).

Thus, the gate delay can be expressed through the following:

$$T_{\text{delay}} = T_{\text{actuation}} + T_{\text{snap-thru}} \tag{11}$$

$T_{\text{snap-thru}}$ is the time period of the bistable beam as it transitions from a critical point snapping to another equilibrium state (*26*). We characterized the $T_{\text{snap-thru}}$ of our bistable beam as about 0.11 s.

Supplementary Note 8 Scaling analysis of the gate delay of OMS

As Supplementary Equation (11) suggests, the lower bound of the gate delay is determined by both $T_{\text{actuation}}$ and $T_{\text{snap-thru}}$. $T_{\text{actuation}}$ is mainly attributed to actuators. To reduce $T_{\text{actuation}}$, we can increase actuation speed. For example, in our case, we can increase the supply power of the CSCP actuator as shown in Fig. 2c. Otherwise, we can instead utilize intrinsically fast actuators, e.g., dielectric elastomer actuators. However, to obtain a smaller gate delay, it is necessary to speed up the snapping duration, which is mainly affected by the geometry and material properties of the bistable beam. According to (*26*), we can estimate the timescale of snapping by

$$T_{*} = \frac{{L_0}^2}{h}\sqrt{\frac{3\rho}{E}} \tag{12}$$

where L_0, h, ρ, and E are the length, thickness, density, and Young's modulus of the beam, respectively. This equation suggests that the most efficient method to reduce $T_{\text{snap-thru}}$ is to use a shorter beam; while increasing thickness, using lightweight or stiffer materials of the beam also help.

Supplementary Note 9 Comparing origami- and semiconductor-based control of the flytrap-inspired robot.

The flytrap-inspired robot with origami-based control is built as described in Supplementary Note 2. To conduct a head-to-head comparison, we created a semiconductor-based traditional analog as shown in Supplementary Fig. 16. The main difference from the origami-based robot is that we used common and easily accessible off-the-shelf semiconductor-based components for control, including a micro-controller (ESP8266), two capacitive touch sensors (TTP223B), and two linear motor modules. Each motor module consists of a servo motor (FS90R), a spool (made of a servo arm), and nylon string. When the motor is activated, it will rotate and drive the spool to contract the nylon string to close the leaves.

The detailed components, weight, and cost of these two robots are listed in Supplementary Table 3. Here we consider the linear motor module as one component although it is composed of three sub-components. Therefore, our origami-based robot only shows a slight advantage over the traditional one on the component count. This comparison result remains valid for most electrically/thermally driven actuators (e.g., SMA) that do not require additional associated components to close the leaves. It is worth noting that these actuators are usually more expensive and less accessible. For more general cases, we often need auxiliary components to convert the motion of actuators to achieve the closure of leaves. For example, when we use linear servo motors instead, specific transmission mechanisms or connectors are required, which increases the component number and thus the complexity.

Our origami-based robot has obvious advantages in terms of weight and cost. Specifically, the traditional robot weighs 2.5 times heavier than the origami one. The ratio could be even larger by replacing the 3D printed supports with lightweight origami counterparts. However, this advantage in weight could be mitigated when including heavy auxiliary components, e.g., batteries. As for cost comparison, we only calculate the total cost of off-the-shelf components. For example, the cost of a capacitive touch sensor is $ 0.84 (from Amazon) while that of an origami AND gate is about $ 0.34, estimated as the total cost of the required PET sheet and copper tape (from Amazon).

In addition, to compare the robustness, we operated these two robots under four different adversarial environmental events, i.e., static magnetic field, RF signal, electrostatic discharge (ESD), and mechanical deformation. (1) Static magnetic field. We used permanent magnet beads to introduce a magnetic field (0.47 T). On the traditional robot, the micro-controller and sensors were not affected by the magnetic field but the motors failed to respond. Upon removal, the traditional robot recovered its functionality. The origami-based one behaved as designed (see Supplementary Movie 9). (2) RF signal. The RF signal around the traditional robot trig-

gered unexpected motor operation. This caused one of the leaves to close, which led to unsuccessful operation after the RF signal was removed. The origami-based counterpart remains intact (see Supplementary Movie 10). (3) ESD. A piezo igniter was adapted to generate high-voltage ESD, which damaged the micro-controller while having no impact on the origami AND gate (see Supplementary Movie 11). (4) Through bending and twisting, we applied mechanical strain rather than stress to the controllers, resulting in failure of the micro-controller but not the origami one (see Supplementary Movie 12). It is worth noting that the pliers were electrically insulated to avoid causing a short circuit.

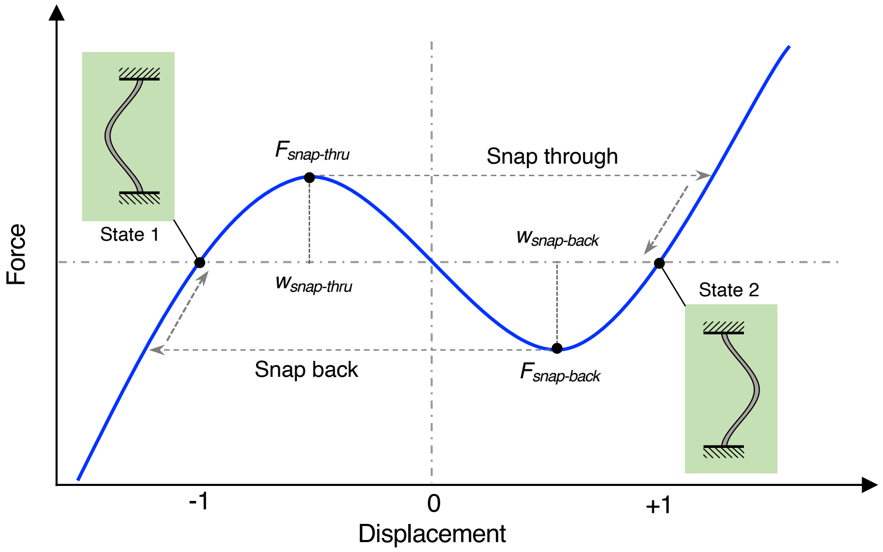

Supplementary Fig. 1. Mechanism of a bistable buckled beam. The bistable beam has two symmetric stable states, i.e., state 1 and state 2. The centerpoint of the beam will snap through towards state 2 when its displacement exceeds $w_{\text{snap-thru}}$. Similarly, the beam will snap back state 1 when it is actuated to $w_{\text{snap-back}}$.

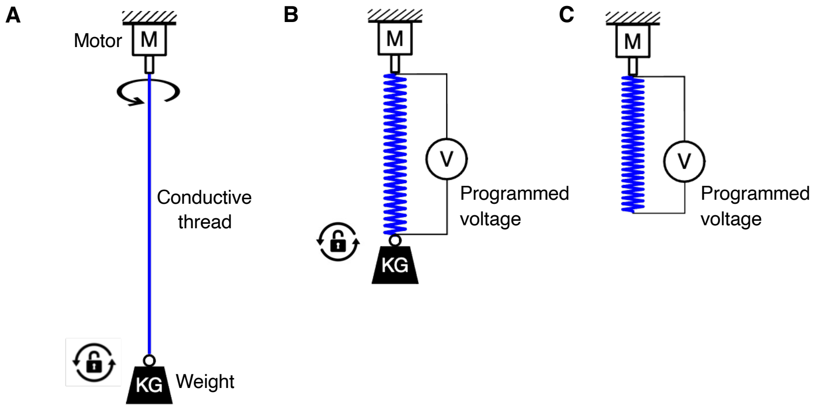

Supplementary Fig. 2. Fabrication of the CSCP actuator. (A) Coils insertion. The conductive yarn was pretensioned by a weight and then twisted by a stepper motor until coils were formed. (B) Annealing process of the coiled yarn. The coiled yarn was periodically heated/cooled to release the twisting stress, resulting in a CSCP actuator. (C) Stabilization process of the actuator. The weight was removed and the actuator was treated by a similar heating/cooling process to stabilize the strain.

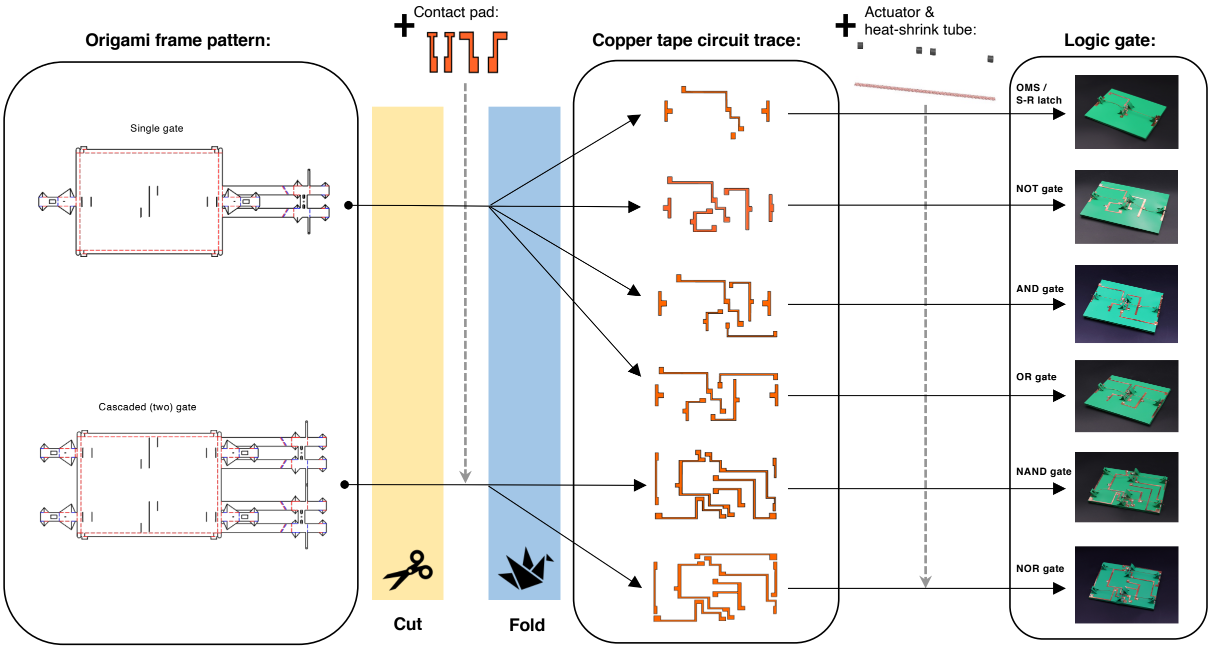

Supplementary Fig. 3. A generalized process of creating OMS-based devices. The fabrication process includes three major steps: (i) cut-and-fold 2D pattern to create origami frame; contact pads are attached on the bistable beam before folding, (ii) affix the copper circuit traces onto the base of the folded frame, and (iii) install CSCP actuator(s) and complete the circuit of the device.

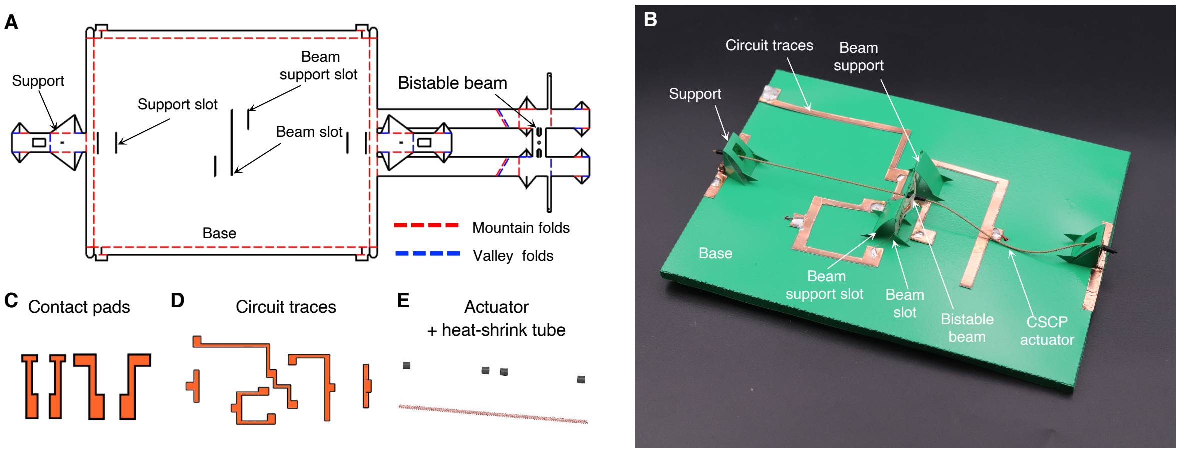

Supplementary Fig. 4. Design and assembly of the NOT gate. (A) 2D pattern of the origami frame. Red dashed lines represent mountain folds and blue lines are valley folds. (B) Assembled origami NOT gate. (C) Contact pads. (D) Circuit traces made of copper tape. (E) CSCP actuators and heat-shrinking tubes.

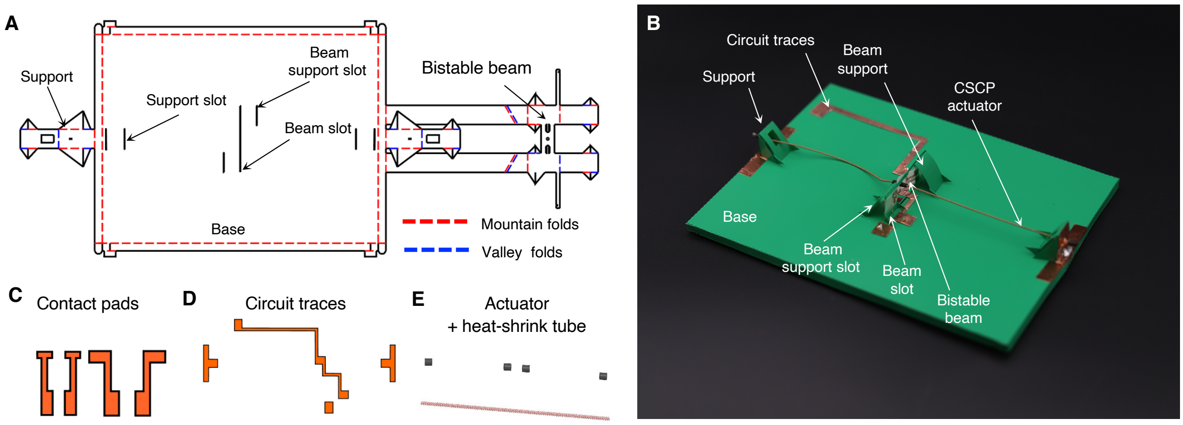

Supplementary Fig. 5. Design and assembly of the OMS / S-R latch. (A) 2D pattern of its origami frame. (B) Assembled origami OMS / S-R latch. (C) Contact pads. (D) Circuit traces. (E) CSCP actuators and heat-shrinking tubes.

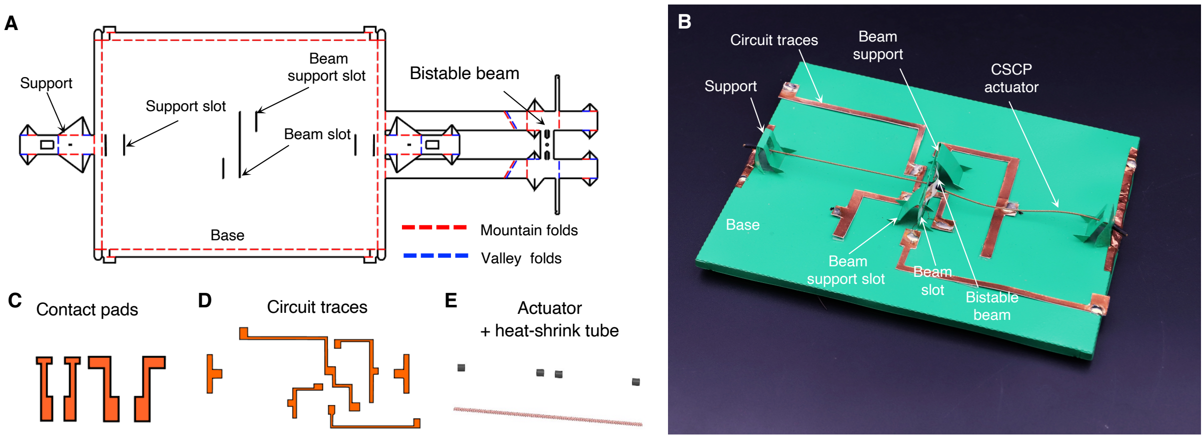

Supplementary Fig. 6. Design and assembly of the AND gate. (A) 2D pattern of the origami frame. (B) Assembled origami AND gate. (C) Contact pads. (D) Circuit traces. (E) CSCP actuators and heat-shrinking tubes.

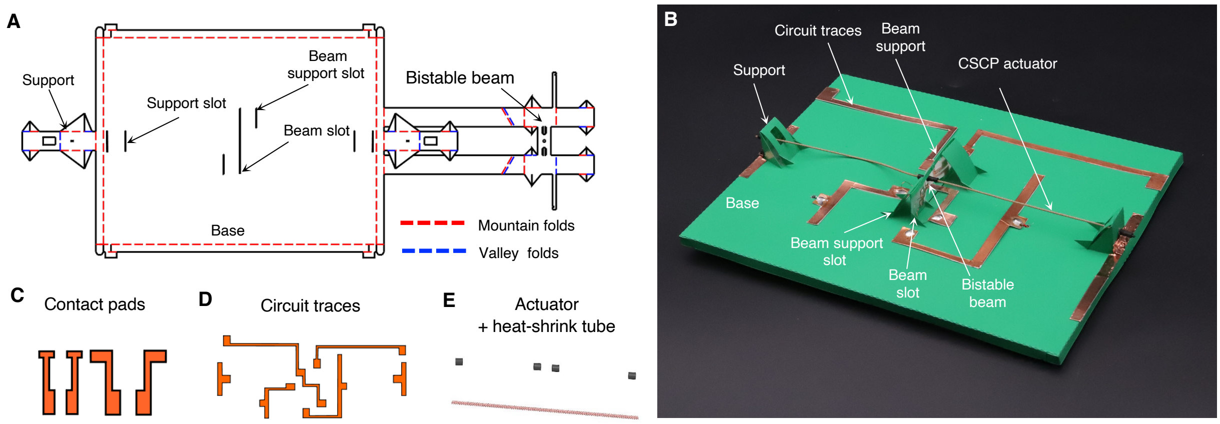

Supplementary Fig. 7. Design and assembly of the OR gate. (A) 2D pattern of the origami frame. (B) Assembled origami OR gate. (C) Contact pads. (D) Circuit traces. (E) CSCP actuators and heat-shrinking tubes.

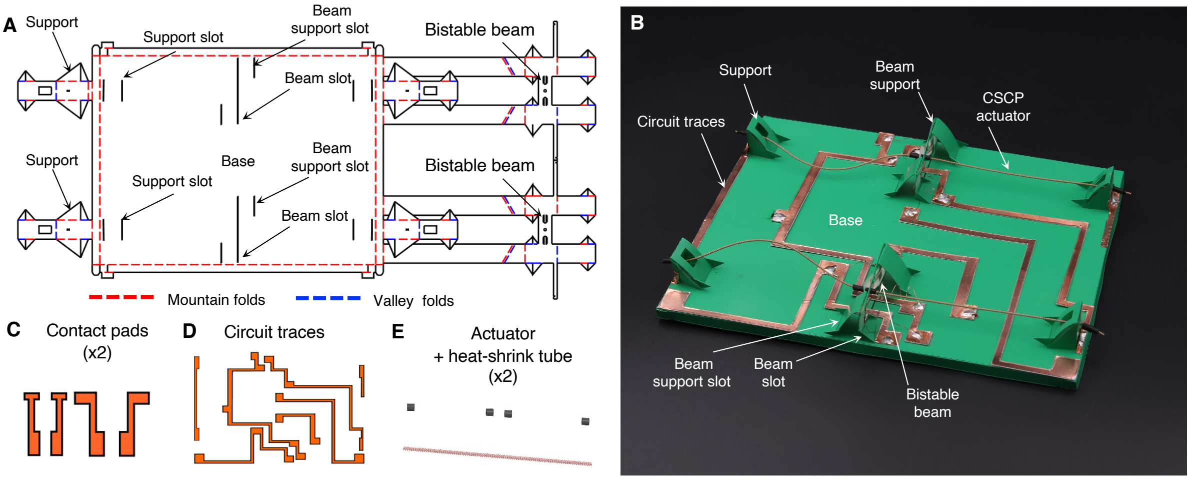

Supplementary Fig. 8. Design and assembly of the NAND gate. (A) 2D pattern of the origami frame. (B) Assembled origami NAND gate. (C) Contact pads. (D) Circuit traces. (E) CSCP actuators and heat-shrinking tubes.

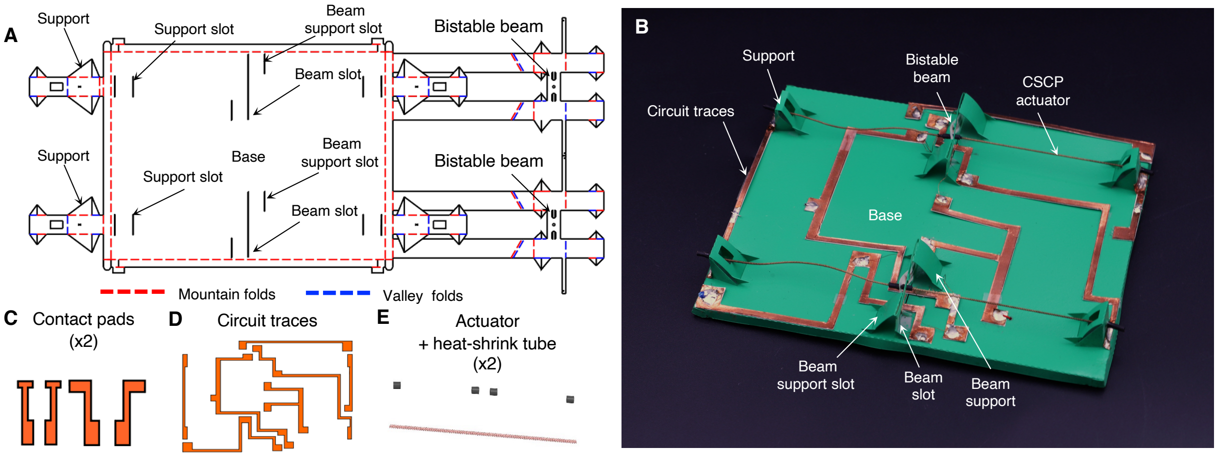

Supplementary Fig. 9. Design and assembly of the NOR gate. (A) 2D pattern of the origami frame. (B) Assembled origami NOR gate. (C) Contact pads. (D) Circuit traces. (E) CSCP actuators and heat-shrinking tubes.

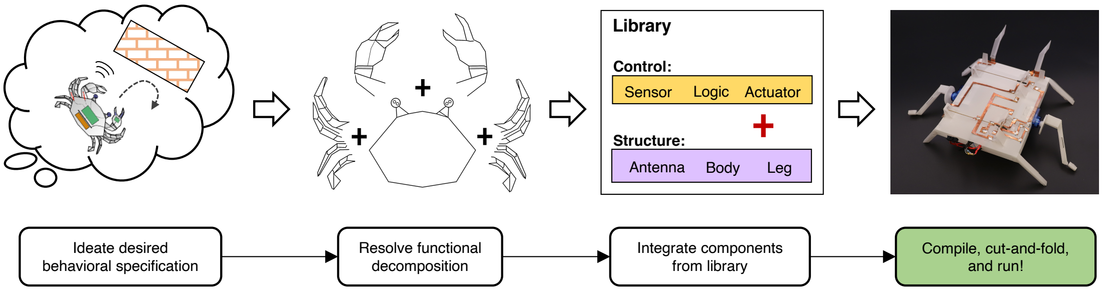

Supplementary Fig. 10. Conceptual workflow to create a functional robot/system using our monolithic paradigm. The capabilities of our manufacturing process could enable a possible computational design pipeline to take as input a schematic outlining a high-level breakdown of the required components and output manufacturable drawings ready for fabrication and assembly. Currently, our system relies on experts to decompose functionalities, combine components from the library, and generate manufacturable drawings for assembly. Further development on automated tools could enable on-demand design for non-expert users: with our existing available components (sensors, switches, logic, controllers, and actuators) as building blocks, users could compose their targeted functionalities and realize the creation of desired robotic devices through folding from functional sheet and thread materials.

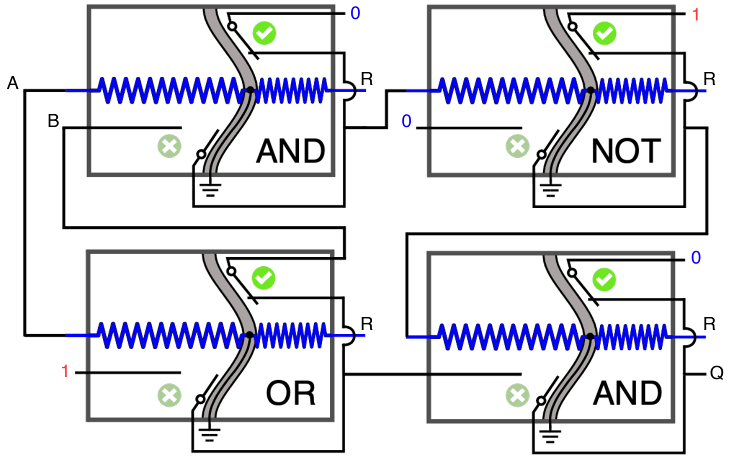

Supplementary Fig. 11. The schematic of origami XOR gate based on OMSs. The XOR gate consists of two AND gates, one OR gate, and one NOT gate.

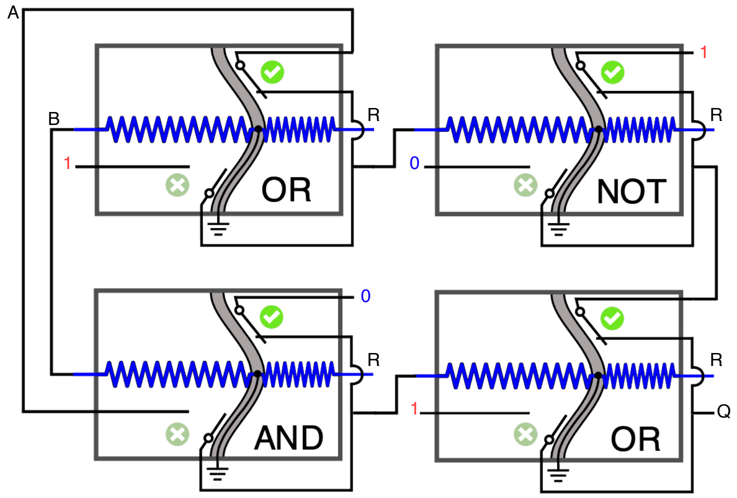

Supplementary Fig. 12. The schematic of origami XNOR gate based on OMSs. The XNOR gate consists of two OR gates, one AND gate, and one NOT gate.

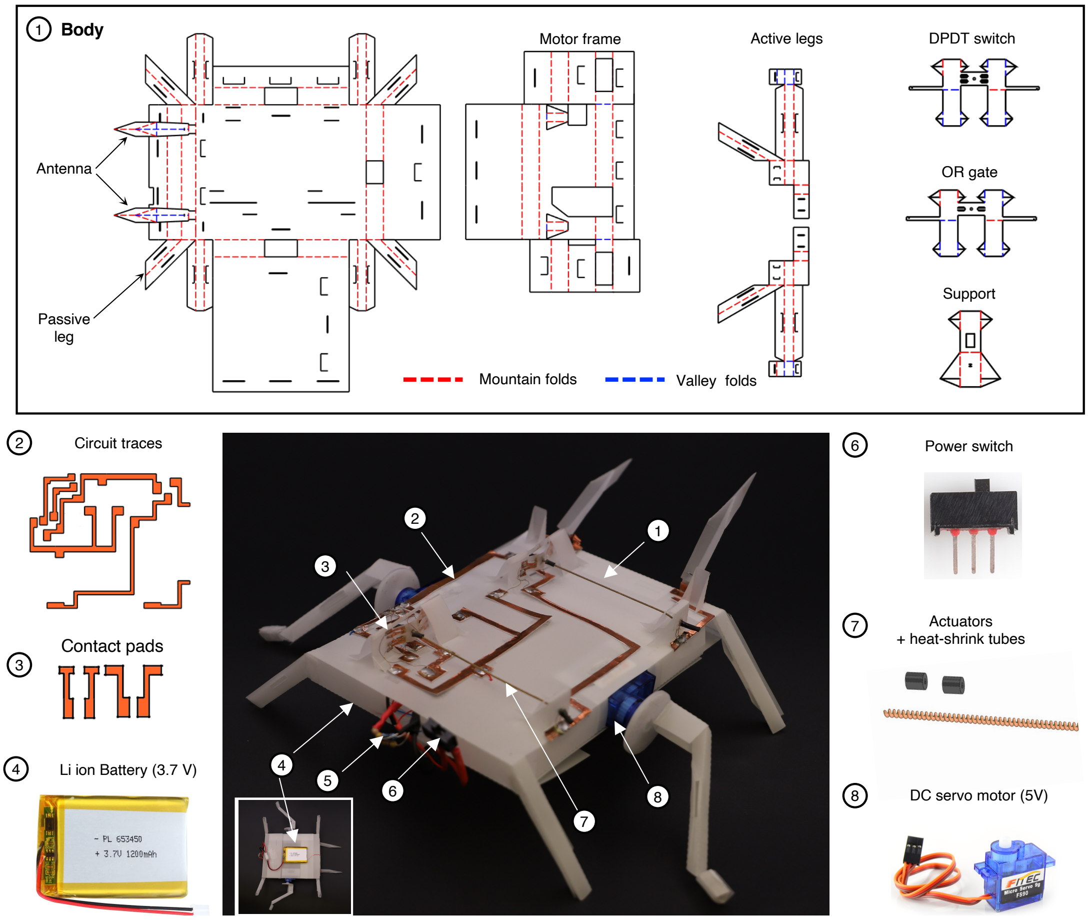

Supplementary Fig. 13. Design and assembly of the legged robot. 2D pattern of the origami structures and a list of necessary components are presented.

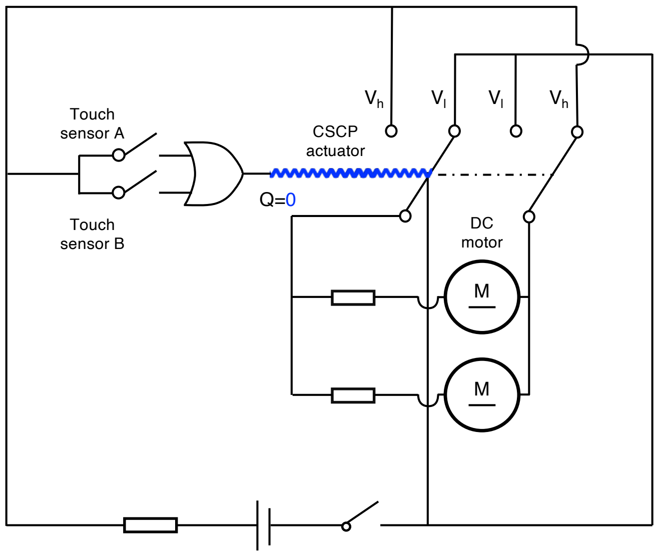

Supplementary Fig. 14. Circuit diagram of the legged robot. Two touch sensors perceive external information and send that as a voltage signal to the OR gate; the output (voltage) of the gate, in turn, is used to activate the CSCP actuator that are used to change the rotation direction of DC motors through a DPDT switch.

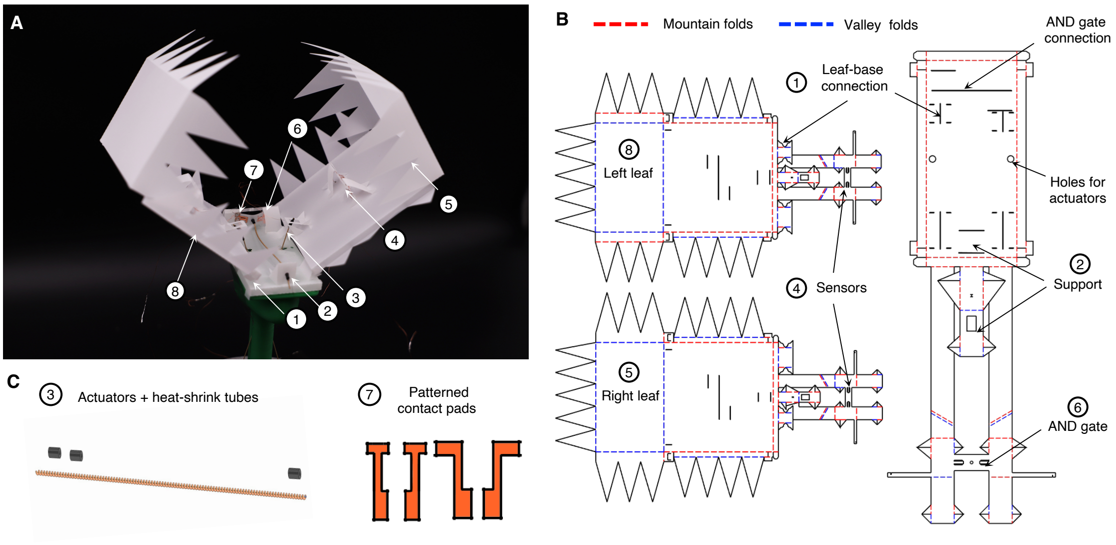

Supplementary Fig. 15. Design and assembly of the flytrap-inspired prey-catching robot. (A) The detailed structure of the robot with labels. (B) 2D fabrication pattern of the origami body of the robot. (C) CSCP actuators and contact pads.

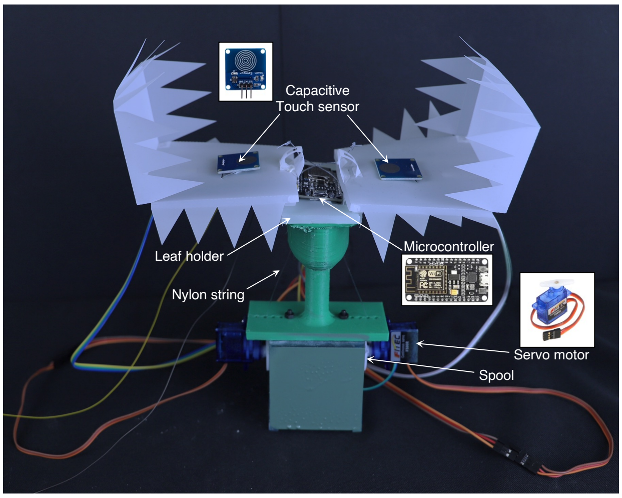

Supplementary Fig. 16. Design and assembly of the traditional flytrap-inspired robot with semiconductor-based control. We built this traditional robot to compare with the origami-based counterpart in terms of complexity, cost, weight, and robustness to adversarial environmental events.

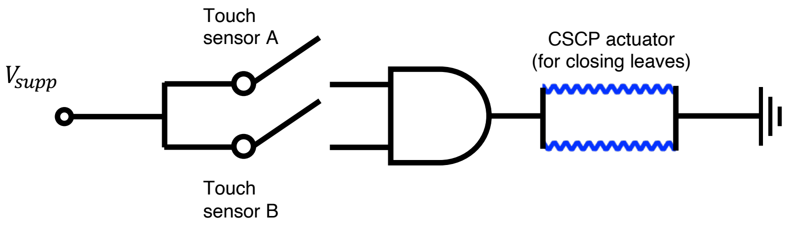

Supplementary Fig. 17. Circuit diagram of the flytrap-inspired prey-catching robot. The information from the two touch sensors on the leaves determines the output of the origami AND gate; its output is used to decide the actuation of the two CSCP actuators, which could contract to close the leaves.

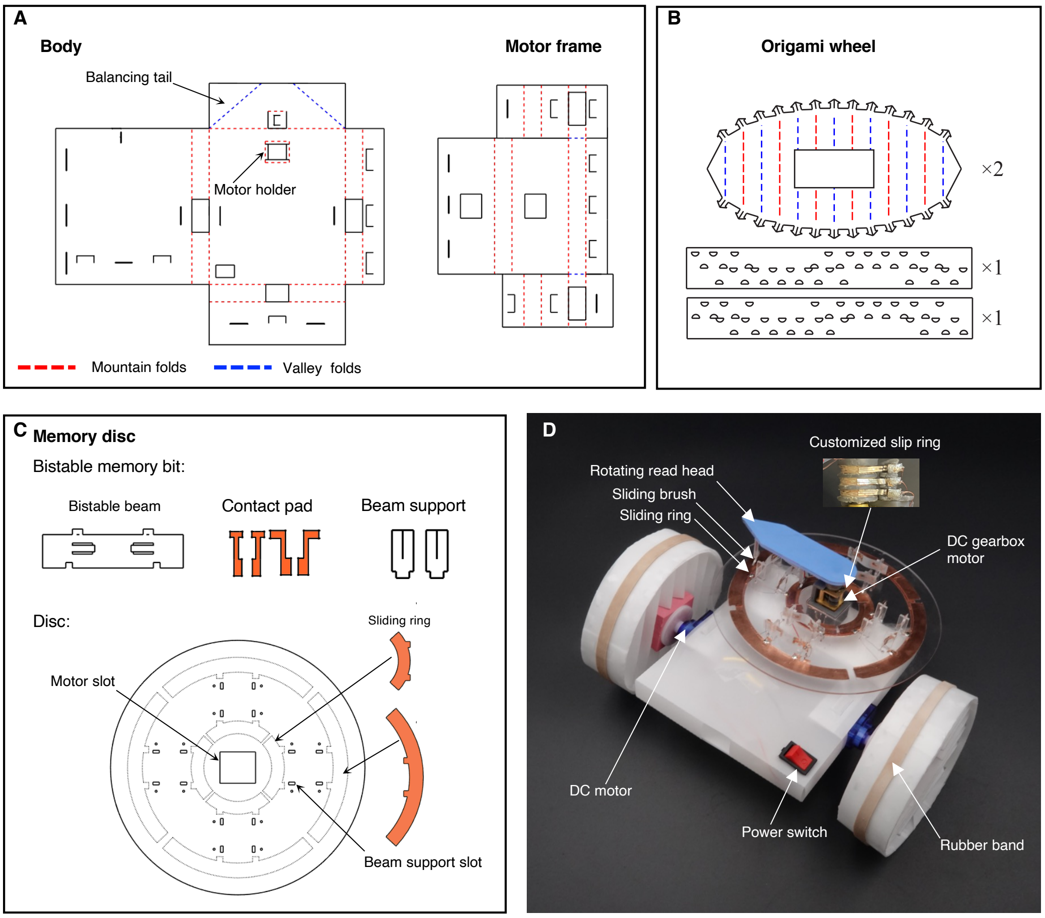

Supplementary Fig. 18. Design and assembly of the origami car with reprogrammable trajectories. (A) 2D fabrication patterns of the origami body with motor frame. (B) The 2D pattern of the origami corrugated wheel. (C) Design of the memory disc. (D) The detailed structure of the car with labels.

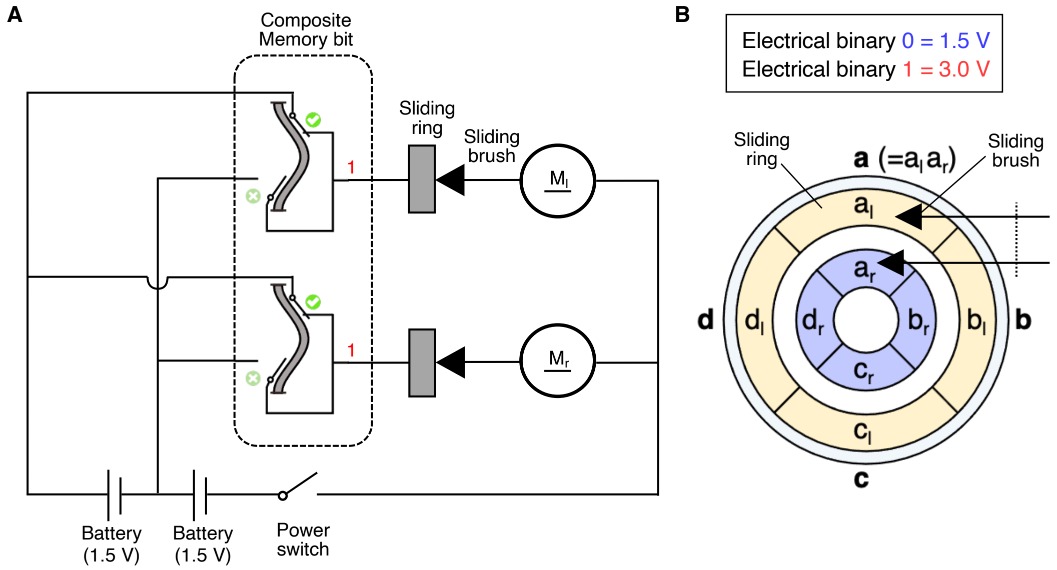

Supplementary Fig. 19. Circuit diagram of the origami wheeled car. (A) An example of the circuit of the car when the head reads a memory of 11. (B) The read head extracts information from **a** to **d** while rotating.

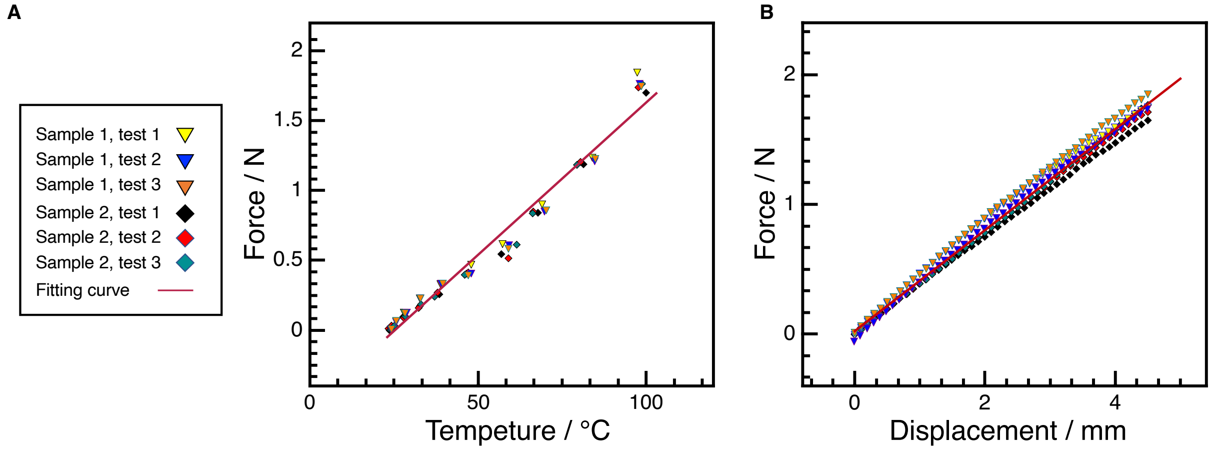

Supplementary Fig. 20. Characterization of the CSCP actuator. (A) The exerted force of the actuator as a function of its temperature when heated up through Joule heating. (B) The force-displacement curve of the actuator in room temperature.

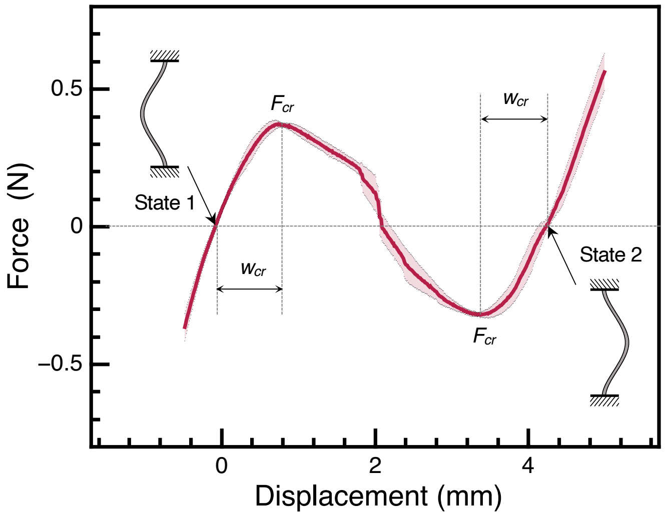

Supplementary Fig. 21. Characterization of the bistable beam. The force-displacement curve of the bistable beam. The shaded area represents standard deviations obtained from three separate experiments.

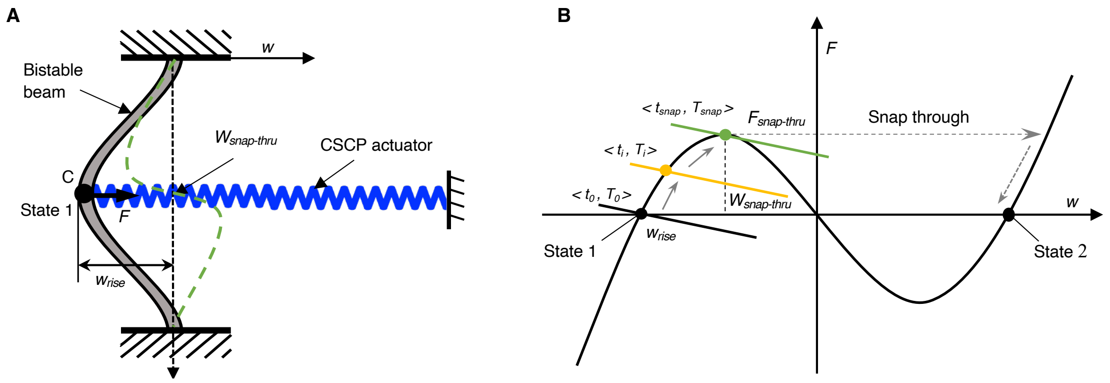

Supplementary Fig. 22. Schematic of the OMS. (A) A simplified model of OMS. The actuation force F from the actuator was applied to the centerpoint C of the bistable beam with an initial rise w_{rise}. The green dashed curve represents the transition mode (from state 1 to state 2) of the bistable beam. (B) The mechanism of the decoupled model of OMS. Black profile is the force-displacement curve of the bistable beam; straight lines represent the force-displacement curves of the CSCP actuator at different moments with their corresponding temperatures.

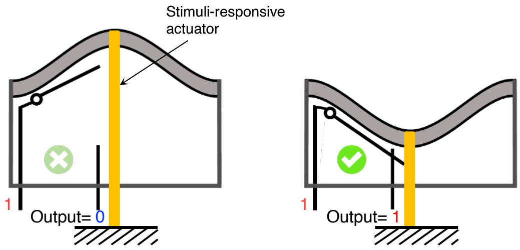

Supplementary Fig. 23. Schematic of sensors for other stimuli. The stimuli-responsive actuators can activate the bistable beam to change the on/off state of the circuit upon perceiving corresponding signals. Thus, various stimuli could be detected and converted to electrical signals to interface with our control units.

Supplementary Table 1. The set of parameters of the CSCP actuator and bistable beam for characterization.

Parameters	Unit	Value
$CSCP\ actuator$:		
Length (x_0)	mm	50.0
Diameter (D)	mm	0.71
Resistance (R)	ohm	2.79
$Bistable\ beam$:		
Length (L_0)	mm	14.9
Width (b)	mm	7.0
Thickness (h)	mm	0.127
Precompression (d_0)	mm	0.9

Supplementary Table 2. Design space of our proposed method.

Components	Implementation	Challenges
Computation :		
Single logic gates (NOT, OR, AND)	✓ (Fig. 3a, c)	
Cascaded logic gates (NOR, NAND)	✓ (Fig. 3d)	
Clock	✓ (Ref (*20*))	
Complex combinational logic	✣	Fabrication
Sequential logic	✣	Fabrication,<br>Integration with registers
Sensing :		
Sensor (touch)	✓ (Fig. 5d, Fig. 6a)	
Sensor (vision)	✗	No available transducers
Sensor (other stimuli)	✣ (Supp. Fig.23)	Need stimuli-responsive materials
Actuation :		
Actuator (electrical/thermal driven)	✓ (Fig. 3b, Fig. 6b)	
Actuator (other stimuli)	✣	Require functional materials
Actuator (linear)	✓ (Fig. 3b, e)	
Actuator (small-angle rotating)	✓ (Fig. 5e)	
Actuator (continuous rotating)	✗	No available rotating mechanism
Memory :		
S-R latch	✓ (Fig. 4a)	
Multi-bit nonvolatile memory	✓ (Fig. 7b)	
Addressable memory bank	✣	Fabrication,<br>Require indexing mechanisms
Power :		
Battery	✣ (Ref (*17, 18*))	Fabrication,<br>Need suitable electrochemical materials

Note: ✣ represents that the component has not been created but it is possible based on our proposed method.

Supplementary Table 3. Comparison between semiconductor- and origami-based control for the flytrap-inspired robot[1].

	Semiconductor-based	Origami-based
Component count [2]:	Total 9	Total 8
Sensing	Capacitive touch sensor (2)	Origami touch sensor (2)
Control	Microcontroller (1)	Origami AND gate (1)
Actuation	Linear motor module[3] (2)	CSCP actuator (2)
Structure	Leaf (2), support (1)	Leaf (2), support (1)
	Leaf holder (1)	
Weight (g):	Total 69.62	Total 28.03
Sensing	Capacitive touch sensor (1.91×2)	Origami touch sensor (0.34×2)
Control	Microcontroller (7.93×2)	Origami AND gate (1.91×2)
Actuation	Linear motor module (12.39×2)	CSCP actuator (0.07×2)
Structure	Leaf (3.13×2), support (17.13)	Leaf (3.13×2), support (17.13)
	Leaf holder (0.77)	
Cost ($) [4]:	Total 19.17	Total 1.48
Sensing	Capacitive touch sensor (0.84×2)	Origami touch sensor (0.08×2)
Control	Microcontroller (5.50)	Origami AND gate (0.32)
Actuation	Linear motor module (5.61×2),	CSCP actuator (0.12×2)
Structure	Leaf (0.02×2), support (0.72)	Leaf (0.02×2), support (0.72)
	Leaf holder (0.01)	
Robustness:		
Magnetic field (0.47 T)	✗	✓
RF signal (power, 5 W)	✗	✓
ESD ($V_{out} \geqslant$ 20kv)	✗	✓
Mechanical deformation	✗	✓

Note: 1. We do not include wiring and power supplies for a fair comparison. 2. We use component count to partially represent the complexity of the resulting robots. 3. Each linear motor module consists of a servo motor, a spool, and a nylon string. However, we consider each module as one component. 4. It is difficult to compare costs accurately. Here we only calculate the total cost of off-the-shelf components to represent the comparison.

Supplementary Table 4. Taxonomy of autonomous origami and soft robots[1].

		Robustness						
Robot	Sense-decide-act loop	Magnetic filed	RF	ESD	Deformation	Easy to fabricate	Lightweight	Low cost[2]
Origami robots:								
Our robot	✓	✓	✓	✓	✓	✓	✓	✓
Ref. (*27*)	✓	✗	✗	✗	✗	✓	✓	✗
Millirobot (*28*)	✓	✓	✗	✗	✗	✗	✓	✓
Ref. (*7*)	✗	✓	✓	✓	✓	✓	✓	✓
Ref. (*29*)	✗	✓	✓	✓	✓	✓	✓	✓
Ref. (*30*)	✗	✓	✓	✓	✓	✗	✓	✗
Ref. (*12*)	✗	✗	✓	✓	✗	✗	✗	✗
Soft robots[3]:								
Ref. (*31*)	✗	✓	✓	✓	✓	✗	✗	✓
Trevor (*32*)	✗	✓	✓	✓	✗	✗	✓	✓
Ref. (*6*)	✓	✓	✓	✓	✓	✗	✗	✓
Ref. (*5*)	✓	✓	✓	✓	✓	✗	✗	✓
Ref. (*33*)	✗	✓	✓	✓	✓	✗	✓	✓
Octobot (*11*)	✗	✓	✓	✓	✓	✓	✓	✓
DEAnsect (*8*)	✓	✓	✗	✗	✓	✗	✓	✓

Note: 1. Here, we only list representative robots that can operate autonomously without requiring external control. These robots can either be powered by onboard supplies or tethered to off-board energy sources. 2. We only consider the cost of off-the-shelf materials. 3. Most remaining autonomous soft robots are composed of semiconductor-based micro-controllers and/or electromagnetic motors, which leads to low robustness against the listed adversarial environmental events and presumably loses their advantages in weight and cost.

Supplementary References

1. Hong, J., Yan, W., Ma, Y., Zhang, D., and Yang, X., Experimental investigation on the vibration tuning of a shell with a shape memory alloy ring, *Smart Mater. Struct.* **24**, 105007 (2015).

2. He, Q., Wang, Z., Wang, Y., Minori, A., Tolley, M. T., and Cai, S., Electrically controlled liquid crystal elastomer–based soft tubular actuator with multimodal actuation, *Sci. Adv.* **5**, eaax5746 (2019).

3. Yasuda, H., Buskohl, P. R., Gillman, A., Murphey, T. D., Stepney, S., Vaia, R. A., and Raney, J. R., Mechanical computing, *Nature* **598**, 39–48 (2021).

4. Yang, D., Verma, M. S., So, J.-H., Mosadegh, B., Keplinger, C., Lee, B., Khashai, F., Lossner, E., Suo, Z., and Whitesides, G. M., Buckling pneumatic linear actuators inspired by muscle, *Adv. Mater. Technol.* **1**, 1600055 (2016).

5. Preston, D. J., Rothemund, P., Jiang, H. J., Nemitz, M. P., Rawson, J., Suo, Z., and Whitesides, G. M., Digital logic for soft devices, *Proc. Natl. Acad. Sci. U.S.A.* **116**, 7750–7759 (2019).

6. Garrad, M., Soter, G., Conn, A., Hauser, H., and Rossiter, J., A soft matter computer for soft robots, *Sci. Robot.* **4**, eaaw6060 (2019).

7. Yan, W. and Mehta, A., A cut-and-fold self-sustained compliant oscillator for autonomous actuation of origami-inspired robots, *Soft Robot.* **9**, 871-881 (2022).

8. Ji, X., Liu, X., Cacucciolo, V., Imboden, M., Civet, Y., El Haitami, A., Cantin, S., Perriard, Y., and Shea, H., An autonomous untethered fast soft robotic insect driven by low-voltage dielectric elastomer actuators, *Sci. Robot.* **4**, eaaz6451 (2019).

9. Wan, G., Liu, Y., Xu, Z., Jin, C., Dong, L., Han, X., Zhang, J. X., and Chen, Z., Tunable bistability of a clamped elastic beam, *Extreme Mech. Lett.* **34**, 100603 (2020).

10. Rus, D. and Tolley, M. T., Design, fabrication and control of origami robots, *Nat. Rev. Mater.* **3**, 101–112 (2018).

11. Wehner, M., Truby, R. L., Fitzgerald, D. J., Mosadegh, B., Whitesides, G. M., Lewis, J. A., and Wood, R. J., An integrated design and fabrication strategy for entirely soft, autonomous robots, *Nature* **536**, 451–455 (2016).

12. Felton, S., Tolley, M., Demaine, E., Rus, D., and Wood, R., A method for building self-folding machines, *Science* **345**, 644–646 (2014).

13. Miyashita, S., Meeker, L., Tolley, M. T., Wood, R. J., and Rus, D., Self-folding miniature elastic electric devices, *Smart Mater. Struct.* **23**, 094005 (2014).

14. Whitney, J. P., Sreetharan, P. S., Ma, K. Y., and Wood, R. J., Pop-up book MEMS, *Journal of Micromech. Microeng.* **21**, 115021 (2011).

15. Kent, T. A., Ford, M. J., Markvicka, E. J., and Majidi, C., Soft actuators using liquid crystal elastomers with encapsulated liquid metal joule heaters, *Multifunct. Mater.* **3**, 025003 (2020).

16. Mehta, A. M., DelPreto, J., Wong, K. W., Hamill, S., Kress-Gazit, H., and Rus, D., Robot creation from functional specifications, *Robotics Research: Volume 2* pp. 631–648 (2018).

17. Wang, M., Vecchio, D., Wang, C., Emre, A., Xiao, X., Jiang, Z., Bogdan, P., Huang, Y., and Kotov, N. A., Biomorphic structural batteries for robotics, *Sci. Robot.* **5**, eaba1912 (2020).

18. Duduta, M., de Rivaz, S., Clarke, D. R., and Wood, R. J., Ultra-lightweight, high power density lithium-ion batteries, *Batter. Supercaps* **1**, 131–134 (2018).

19. Song, Z., Ma, T., Tang, R., Cheng, Q., Wang, X., Krishnaraju, D., Panat, R., Chan, C. K., Yu, H., and Jiang, H., Origami lithium-ion batteries, *Nat. Commun.* **5**, 1–6 (2014).

20. Yan, W., Gao, A. L., Yu, Y., and Mehta, A., Towards autonomous printable robotics: Design and prototyping of the mechanical logic, *Proceedings of the 2018 International Symposium on Experimental Robotics* pp. 631–644 (2020).

21. Yan, W. and Mehta, A., Towards one-dollar robots: An integrated design and fabrication strategy for electromechanical systems, *Robotica* **41**, 31–47 (2023).

22. Liu, C., Yan, W., and Mehta, A., Computational design and fabrication of corrugated mechanisms from behavioral specifications, *2021 IEEE International Conference on Robotics and Automation (ICRA)* pp. 7087–7093 (2021).

23. Yip, M. C. and Niemeyer, G., On the Control and Properties of Supercoiled Polymer Artificial Muscles, *IEEE Trans. Robot.* **33**, 689–699 (2017).

24. Nayfeh, A. and Ibrahim, R., Nonlinear interactions: analytical, computational, and experimental methods, *Appl. Mech. Rev.* **54**, B60–B61 (2001).

25. Yan, W., Yu, Y., and Mehta, A., Rapid design of mechanical logic based on quasi-static electromechanical modeling, *2019 IEEE/RSJ International Conference on Intelligent Robots and Systems (IROS)* pp. 5820–5825 (2019).

26. Gomez, M., Moulton, D., and Vella, D., Critical slowing down in purely elastic ‘snap-through’ instabilities, *Nat. Phys.* **13**, 142–145 (2017).

27. Onal, C. D., Tolley, M. T., Wood, R. J., and Rus, D., Origami-inspired printed robots, *IEEE/ASME Trans. Mechatronics* **20**, 2214–2221 (2014).

28. Zhakypov, Z., Mori, K., Hosoda, K., and Paik, J., Designing minimal and scalable insect-inspired multi-locomotion millirobots, *Nature* **571**, 381–386 (2019).

29. Yan, W. and Mehta, A., A crawling robot driven by a folded self-sustained oscillator, *2022 IEEE 5th International Conference on Soft Robotics* pp. 455–460 (2022).

30. Kim, S.-W., Koh, J.-S., Lee, J.-G., Ryu, J., Cho, M., and Cho, K.-J., Flytrap-inspired robot using structurally integrated actuation based on bistability and a developable surface, *Bioinspir. Biomim.* **9**, 036004 (2014).

31. Preston, D. J., Jiang, H. J., Sanchez, V., Rothemund, P., Rawson, J., Nemitz, M. P., Lee, W.-K., Suo, Z., Walsh, C. J., and Whitesides, G. M., A soft ring oscillator, *Sci. Robot.* **4**, eaaw5496 (2019).

32. Henke, E.-F. M., Schlatter, S., and Anderson, I. A., Soft dielectric elastomer oscillators driving bioinspired robots, *Soft Robot.* **4**, 353–366 (2017).

33. Drotman, D., Jadhav, S., Sharp, D., Chan, C., and Tolley, M. T., Electronics-free pneumatic circuits for controlling soft-legged robots, *Sci. Robot.* **6**, eaay2627 (2021).
