# Supplementary material for: Origami-based integration of robots that sense, decide, and respond
Source: Nat Commun. 2023 Apr 3;14:1553. doi: 10.1038/s41467-023-37158-9 (PMC10070436; doi:10.1038/s41467-023-37158-9)
Supplement: Supplementary file 3 — Description of Additional Supplementary Files [file 41467_2023_37158_MOESM3_ESM.pdf]

Description of additional supplementary files

File name: Supplementary Movie 1

Description: OMS NOT gate.

File name: Supplementary Movie 2

Description: OMS AND gate.

File name: Supplementary Movie 3

Description: OMS OR gate.

File name: Supplementary Movie 4

Description: OMS cascaded NAND gate.

File name: Supplementary Movie 5

Description: OMS cascaded NOR gate.

File name: Supplementary Movie 6

Description: Untethered legged robot autonomously avoiding obstacles.

File name: Supplementary Movie 7

Description: Flytrap-inspired robot autonomously recognizing and capturing “living preys”.

File name: Supplementary Movie 8

Description: Origami wheeled car with reprogrammable trajectories.

File name: Supplementary Movie 9

Description: Performance of flytrap-inspired robots under a magnetic field.

File name: Supplementary Movie 10

Description: Performance of flytrap-inspired robots with RF signal.

File name: Supplementary Movie 11

Description: Performance of flytrap-inspired robots under electrostatic discharge.

File name: Supplementary Movie 12

Description: Performance of flytrap-inspired robots under mechanical deformation.
